# k-Skip-n-Gram-RF: A Random Forest Based Method for Alzheimer's Disease Protein Identification

**DOI:** 10.3389/fgene.2019.00033

**Published:** 2019-02-12

**Authors:** Lei Xu, Guangmin Liang, Changrui Liao, Gin-Den Chen, Chi-Chang Chang

**Affiliations:** ^1^School of Electronic and Communication Engineering, Shenzhen Polytechnic, Shenzhen, China; ^2^Key Laboratory of Optoelectronic Devices and Systems of Ministry of Education and Guangdong Province, College of Optoelectronic Engineering, Shenzhen University, Shenzhen, China; ^3^Department of Obstetrics and Gynecology, Chung Shan Medical University Hospital, Taichung, Taiwan; ^4^School of Medical Informatics, Chung Shan Medical University, Taichung, Taiwan; ^5^IT Office, Chung Shan Medical University Hospital, Taichung, Taiwan

**Keywords:** Alzheimer's disease, n-gram model, random forest, gene coding, sequence information

## Abstract

In this paper, a computational method based on machine learning technique for identifying Alzheimer's disease genes is proposed. Compared with most existing machine learning based methods, existing methods predict Alzheimer's disease genes by using structural magnetic resonance imaging (MRI) technique. Most methods have attained acceptable results, but the cost is expensive and time consuming. Thus, we proposed a computational method for identifying Alzheimer disease genes by use of the sequence information of proteins, and classify the feature vectors by random forest. In the proposed method, the gene protein information is extracted by adaptive k-skip-n-gram features. The proposed method can attain the accuracy to 85.5% on the selected UniProt dataset, which has been demonstrated by the experimental results.

## Introduction

Alzheimer's disease (AD) is a common cause of dementia, and it can lead a degeneration of brain. The research shows that more than 35 million people have been affected by Alzheimer's disease all over the world. It is predicted that there will be over 70 million people diagnosed by Alzheimer's disease in 2030, and the number will be increased by 50% in 2050 (Brookmeyer et al., 2007).

Until now, there is no treatment for AD. As the status becoming worse, it will destroy the ability of speak and think. At last, AD will lead to die. So, it is meaningful to predict AD at an early stage. Machine learning methods have been extensively used in multiple fields of bioinformatics (Zeng et al., 2014; Wang et al., 2016; Liu Y. et al., 2017; Zhang et al., 2017a; Cheng et al., 2018; Fu et al., 2018; Liu et al., 2018; Peng et al., 2018a; Song et al., 2018), such as anticancer peptides prediction (Xu et al., 2018b), identification of antioxidant proteins (Xu et al., 2018a), disease gene identification (Jiang et al., 2017; Liu G. et al., 2017; Peng et al., 2017; Zeng et al., 2017a; Zhang et al., 2017b; Cheng et al., 2018,a,b; Liu et al., 2018a,b; Zhu et al., 2018), microRNA classification (Wei et al., 2014; Chen et al., 2016; Zeng et al., 2018; Zhang et al., 2018), protein remote homology detection (Liu et al., 2014b; Liu and Li, 2018), drug-induced hepatotoxicity prediction (Li et al., 2018; Su et al., 2018), DNA binding protein identification (Zhang and Liu, 2017; Liu, 2018), protein interaction identification (Guo et al., 2011, 2012, 2014; Ding et al., 2016, 2017a,b; Peng et al., 2018b) and so on (Li et al., 2016, 2017; Zou et al., 2016; Zeng et al., 2017a,b; Hu et al., 2018; Xue et al., 2018; Zhang and Liu, 2018; Zhang et al., 2018). In this paper, machine learning method is used to identify AD.

**Figure 1:**
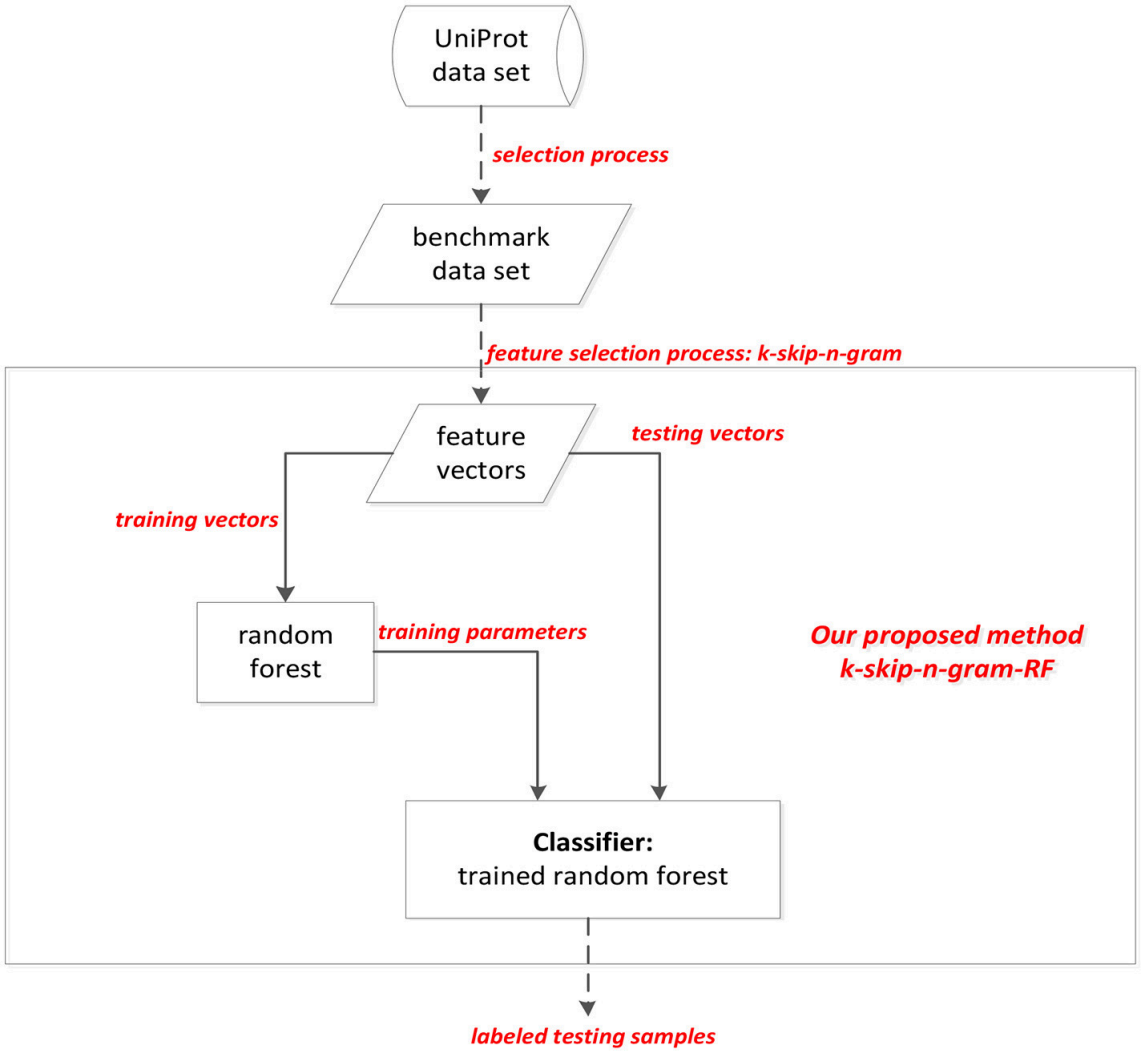
The flow chart of proposed method.

Because the structural features of brain is related to AD, the structural brain information is described by structural magnetic resonance imaging (MRI) data. Most existing works use machine learning methods, such as ensemble classifier, deep learning method to classify AD and non-AD samples. However, most existing works are limited by the expensive cost on money and time. For the purpose of identifying AD efficiently and effectively, a method called k-skip-n-gramRF, which is based on gene coding information of proteins, is proposed to recognize AD samples. In this paper, adaptive k-skip-2-gram is used to extract the information from the protein sequences, and then the samples are classified by random forest (RF) classifier. Consequently the classification accuracy can attain the accuracy to 85.5% using the select data set from Uniprot database. In our proposed method, adaptive k-skip-n-gram describes the correlation information of both adjacent and non-adjacent residues based on traditional n-gram model (Wei et al., 2017a). The idea of our proposed method is shown in Figure 1. As Figure 1 shown, the protein peptides are extracted by k-skip-n-gram method. Each sequence is transferred into a vector. The training vectors are used to train the parameters of random forest. The performance of methods is evaluated by testing vectors. The testing vectors are labeled by trained random forest classifier.

In the proposed method, adaptive n-gram-k-skip model is used to represent the gene coding information by a 400-dimensional vector. Then an ensemble classifier named random forest (RF) is used to classify the samples. In the experiments, the accuracy of the proposed classifier is 85.5%, which is competitive to existing works with low cost. In other words, the experimental results demonstrated that the proposed methods can be utilized to identify AD samples. The contributions of our work include:

- A computational model for predicting Alzheimer's disease is proposed in the paper. The experimental results demonstrate that the classification accuracy of the prediction model is 85.5%, which is competitive to some existing works with low cost and fast speed.
- Different from previous work using MRI data, the gene coding information of proteins is considered to identify Alzheimer's disease protein. Each protein sequence is represented by a 400-dimensional vector, which the information of distance is considered.
- In our work, random forest is used to classify the AD protein peptides and non-AD protein peptides. Random forest is an ensemble classification method based on bagging, which is used to predict AD peptides in the work.

The rest of the paper is organized as follows. Section Materials and Methods introduces the dataset and the proposed method (k-skip-n-gram-RF) for identifying AD peptides. The results of AD prediction are described in Section Results and Discussions. The conclusion is made in Section Conclusions.

## Materials and Methods

### Benchmark Dataset

The used data is selected from the UniProt database. The data set ***S***is composed of positive samples ***S***^+^ and negative samples ***S***^−^. The positive sample set is represented by Alzheimeir's disease (AD) samples, and the negative sample set is represented by non-AD samples.

#### Positive Data Set

The positive data set contains AD samples. The samples are built by the sequences which are labeled by “Alzheimer's disease.” As a result, 310 AD samples are selected from the UniProt database. To avoid the overestimation of the performance, the sequences with more than 60% similarity are removed. Thus, there are 279 positive samples left.

#### Negative Data Set

The data labeled with “non Alzheimer's disease” are chosen, then there are 312 non-AD samples. The proteins which are confirmed as non Alzheimer's disease are also selected in the negative data set. After CD-HIT program (Fu et al., 2012), 1,743 negative samples are left in the benchmark data set for experiments.

In the experiments, the benchmark data set is divided into training data set and testing data set. The training data are used for train the classifier, and the testing data are used for the performance evaluation.

### Random Forest

Random forest (Ho, 1995) is an ensemble classifier by combining decision trees together. Due to its effectiveness, random forest has been widely used in many bioinformatics problems (Deng and Chen, 2015; Liu, 2018). The key idea will be introduced briefly here.

The key element in random forest is decision tree. The decision trees are built based on bagging. Bagging is a sampling method. The used samples will be put back into the data set for reusing. In other words, a sample may be used more than one time for building data set. For example, there is a data set with ***n***samples. If ***m***decision trees are needed, ***m***data sets will be built by bagging for training. Each node on the decision tree is represented by a feature used for classification (Quinlan, 1986). The features used on different levels of the tree are selected in sequence by the entropy value. Entropy is considered as information gain, and the entropy is calculated as Equation (1). The information gain is calculated as Equation (2)

(1) $$\begin{array}{l} {\text{E}}_{\text{i}}\left(x\right)=-\sum_{i=1}^{k}p_{i}\left(x\right)\log^{p_{i}\left(x\right)} \end{array}$$

(2) $$\begin{array}{l} \text{EG}=\text{Entropy}-\sum_{\text{x}}{\text{E}}_{\text{i}}\left(\text{x}\right) \end{array}$$

**Figure 2:**
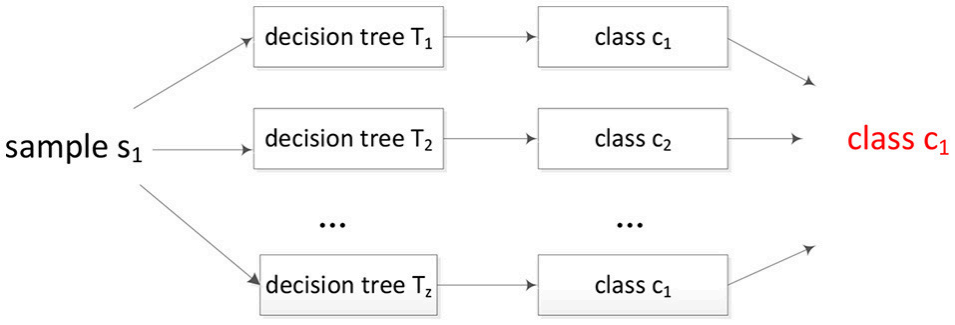
The voting process of random forest.

The attribute with the maximum entropy gain is selected. Random forest is built based on the decision tree, so the features with larger information gains will be selected first in the training process. Because random forest is an ensemble classifier, the decision is made by voting process shown as Figure 2. As Figure 2 shown, the sample will be assigned to the class with the maximum votes. In our problem, the decision trees are trained by protein sequences, and the input of Figure 2 is protein peptide.

### Sequence Representation

The sequence information of each protein peptides is encoded into a 400-dimensional feature vector by adaptive k-skip-2-gram model (Wei et al., 2017a). The k-skip-2-gram method is proposed based on k-skip-n-gram. The key idea of k-skip-n-gram is the distance information integrated into traditional n-gram model (Liu et al., 2014a). However, the maximum value of k is the length of the shortest amino acid, which is small for long peptides. Thus, for the purpose of mining more relation between peptides, adaptive k-skip-n-gram method is proposed in and used in our method.

There is a peptide sequence S, denoted by R_1_R_2_ … R_n_, where n is the length of the sequence. In n-gram model, the occurrence frequencies of any n consecutive amino acids are measured. The amino acid set is denoted as *L*, where *L*_*i*_ is the *i*th element in L. The n-gram features can be calculated as follows:

$${\text{F}}_{\text{n\_gram}}\;=\frac{\text{N}({\text{T}}_{{\text{L}}_{{\text{m}}_{1}}{\text{L}}_{{\text{m}}_{2}}\ldots {\text{L}}_{{\text{m}}_{\text{n}}}})}{\text{N}({\text{T}}_{\text{s}})}$$

Where N(T_s_) is denoted as the number of all items in the set T_s_, and T_s_ represents the number of segments with *n* consecutive amino acids in the peptide S. Each position has 20 possible amino acids, so there are 20^n^ dimensions with the length of n peptides in n-gram model. It is obvious that the features are sparse. Thus, n-gram-k-skip is proposed to overcome the sparse problem of n-gram model. The distance information is considered in k-skip-n-gram model.

In k-skip-n-gram model, distance between residues R_i_ and R_j_ is calculated as Equation (3). For example, if there is *i* = 2 and *j* = 3, the distance between A_2_ and A_3_ is 0.The distance between A_4_ and A_2_ is 1, because A_2_ and A_4_ is separated by A_3_.

(3) $$\begin{array}{l} \text{Dis}=|\text{j}-\text{i}-1| \end{array}$$

In the k-skip-n-gram model, the sequence information of n residues within distance k is calculated, which means that the only residues within distance k are considered. The calculation of k-skip-n-gram is shown as Equation (4). In Equation (4), N(T_SkipG_) is denoted as all the elements in T_SkipG_. The calculation of T_SkipG_ is shown as Equation (5). In Equation (5), Skip(DT = z) = {A_i_A_i+z+1_…A_i+z+n−1_|1 ≤ z ≤ L−1, 1 ≤ z ≤ k}. When n equals 1, the model is reduced to n-gram model. For the purpose of avoiding overfitting problem, n is constrained less than 3. Thus, only the case of n equals to 2 is analyzed. The model is considered as k-skip-2-gram. The elements of k-skip-2-gram include R_1_R_2_,R_2_R_3_,…,R_n−1_R_n_,R_1_R_3_,… R_n−2_R_n_,… R_1_R_n_, which all of them are two amino acids pair within distance k. The number of the 2-item combination is 400. Thus, the number of features extracted by k-skip-2-gram is 400. The 20^n^ dimensional vector is reduced to a 400 dimensional vector.

(4) $$\begin{array}{l} \text{fv}=\left\{\frac{{\text{N}}^{\prime }\left({\text{L}}_{{\text{m}}_{1}}{\text{L}}_{{\text{m}}_{2}}{\ldots \;L}_{{\text{m}}_{\text{n}}}\right)}{\text{N}\left({\text{T}}_{\text{SkipG}}\right)}\right\} \end{array}$$

(5) $$\begin{array}{l} {\text{T}}_{\text{SkipG}}=\left\{\cup_{\text{z}=1}^{\text{k}}\text{Skip}\left(\text{DT}=\text{z}\right)\right\} \end{array}$$

The amino acids distance within k is calculated. K is the minimum sequence length of the peptides. The length of some sequences is sometimes short. If k is small, the features will be limited in local information. In adaptive k-skip-n-gram, k is the length of each sequence. When the information of varying distances of sequences is described, adaptive k-skip-n-gram is more flexible than k-skip-n-gram.

### Performance Evaluation

In the literature of bioinformatics, accuracy(Acc), specificity(Sp), sensitivity(Sn) are frequently used for evaluating the performance of classification methods (Chou, 2001a,b). The performance of the method is measured by the above metrics. Specificity is used to measure the rate of retrieved true positive samples of the real positive samples, which is represented by Equation (6). Sensitivity is the metric for measuring the rate of real non-AD samples identified as non-AD samples of real non-AD samples, which is calculated by Equation (7). Accuracy is the rate that the samples are classified into the correct class, shown as Equation (8).

(6) $$\begin{array}{l} \text{Sp}=\frac{TP}{P^{+}} \end{array}$$

(7) $$\begin{array}{l} \text{Sn}=\frac{TN}{P^{-}} \end{array}$$

(8) $$\begin{array}{l} \text{Acc}=\frac{TP+TN}{P^{+}+P^{-}} \end{array}$$

Where P^+^ is the number of AD samples, and P^−^ is the number of non-AD samples. TP is denoted as the number of AD samples recognized as AD samples. TN is represented by the number of non-AD samples labeled by non-AD samples by the classifier.

## Results and Discussions

In the experiments, the data set is divided into training set and testing set. The training set is used for learning parameters, and the testing set is for performance evaluation. The performance of our proposed method is reported in Section The Performance of Proposed Method. The performance of our method compared with other feature selection methods is described in Section The Comparison of Performance Evaluation on Feature Extraction Methods. We also compared random forest with other classifiers, and the performance evaluation comparison is shown in Section The Comparison of Performance Evaluation on Other Classifiers.

### The Performance of Proposed Method

The experimental results of our proposed method are reported in Table 1. Table 1 shows that the accuracy of our proposed method is 0.855, which means that the proposed method can classified the 85.5% samples correctly in the benchmark data set. Sp(specificity) describes the performance for identifying AD samples. 85.5% AD samples of all the positive samples in the data set will be recognized in the experiment. Moreover, 85.5% non-AD samples of the negative samples can be classified correctly by the proposed method. The experimental results demonstrated that the method is practical.

**Table 1:** The performance evaluation of k-skip-n-gram-RF.

	**Sn**	**Sp**	**Acc**
k-skip-n-gram-RF	0.855	0.855	0.855

### The Comparison of Performance Evaluation on Feature Extraction Methods

For the purpose of showing the effective performance of our proposed method, the feature select method of our proposed method is compared with information theory. Information theory is a feature selection method representing.

The comparison results are shown in Table 2. The metrics of accuracy, sp and sn of k-skip-n-gram method performs are better than that of information theory. The accuracy of the proposed method (k-skip-n-gram-RF) is better than that of information theory based random forest. The accuracy of information theory method is 0.715, while the accuracy of k-skip-n-gram is 0.855. For the problem of Alzheimer's disease protein prediction, k-skip-n-gram performs better than information theory when random forest is used.

**Table 2:** Comparison of our features with other methods on Sn.

	**Sn**	**Sp**	**Acc**
k-skip-n-gram-RF	0.855	0.855	0.855
Information theory-RF	0.714	0.717	0.715

### The Comparison of Performance Evaluation on Other Classifiers

To demonstrate the performance of our classifier, the classification methods are compared with other classification method, such as naive bayes (Peter Norvig, 1995), LibD3C (Lin et al., 2014), Adaboost (Rojas, 2009), and bagging. The mentioned methods are shown as followings.

Naive bayes is probability method. The sample is labeled by the class with the maximum probability.

LibD3C is an ensemble based method. k-means is integrated into the method for classifier selection.

Adaboost and bagging are ensemble classification algorithms. The difference between then is the building strategy of sample set. Bagging reuses the samples during classification. In Adaboost, the samples classified to the wrong class, the weight will be increased. The samples which are classified into the correct class, the weight will be decreased.

**Figure 3:**
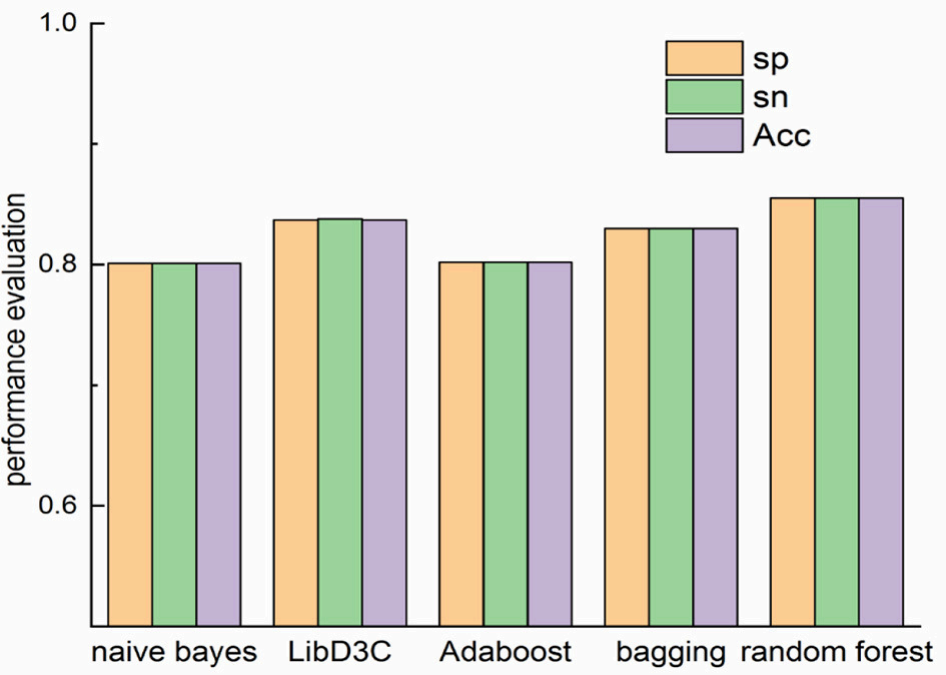
Comparison of performance evaluation on different classifiers.

The comparison of our proposed method with other classifiers on Sn, Sp and accuracy is shown in Figure 3. Random forest performs better than other classifiers on Sn, Sp and Acc. Random forest is an ensemble classifier with competitive performance. The accuracy of naïve bayes and is 0.801 and 0.812. The accuracy of bagging and LibD3C is 0.83 and 0.837. When the features of proteins are extracted by k-skip-n-gram method, the accuracy of random forest is 0.855, which is better than that of other methods. As the results shown, LibD3C preforms better than naïve bayes, Adaboost and bagging.

The feature selection method and classifiers are compared. The k-skip-n-gram method can represent more accurately than information theory. Random forest performs better than other classifiers. Thus, the experimental results demonstrate that our proposed method can provide competitive results.

## Conclusions

In this paper, we study the problem of Alzheimer's disease prediction by using gene coding information. MRI data are usually used to identifying Alzheimer's disease, but the data are complex and with expensive cost. Different from previous work, the information is extracted from the protein peptides by k-skip-n-gram model for Alzheimer's disease prediction. Finally, random forest is used to classify Alzheimer's disease (AD) samples and non-AD samples. The accuracy of our proposed method is 85.5%, which means that it is meaningful for Alzheimer's disease detection with low cost. In the literature, most work have provided web server for protein classification, and we will develop our web server for Alzheimer's disease proteins classification. Moreover, to further improve the prediction performance, there are still many aspects can be explored. For example, other effective machine learning algorithms, such as ensemble learning algorithms and deep learning algorithms, have recently showed they can achieve better performance than traditional algorithms (Mrozek et al., 2009a; Wei et al., 2017b, 2018a; Wang et al., 2018). On the other hand, feature representation learning has demonstrated that it can exploit more informative features and improve the performance in multiple bioinformatics problems (Mrozek et al., 2009b; Momot et al., 2010; Liu et al., 2015; Wei et al., 2018b, 2019; Tang et al., 2019).

## Author Contributions

LX initially drafted the manuscript and did most of the codes work and the experiments. CL and G-DC collected the features, analyzed the experiments and revised the paper. GL and C-CC revised to draft the manuscript. All authors designed the work, read and approved the final manuscript and are agree to be accountable for all aspects of the work.

### Conflict of Interest Statement

The authors declare that the research was conducted in the absence of any commercial or financial relationships that could be construed as a potential conflict of interest.
